# The Collaborative Study on the Genetics of Alcoholism: An Update

**Published:** 2002

**Authors:** Howard J. Edenberg

**Affiliations:** Howard J. Edenberg, Ph.D., is a Chancellor’s Professor in the Department of Biochemistry and Molecular Biology and the Department of Medical and Molecular Genetics, Indiana University School of Medicine, Indianapolis, Indiana

The Collaborative Study on the Genetics of Alcoholism (COGA) is a large-scale family study designed to identify genes that affect the risk for alcoholism (i.e., alcohol dependence) and alcohol-related characteristics and behaviors (i.e., phenotypes^1^). This collaborative project is funded by the National Institute on Alcohol Abuse and Alcoholism. Data collection, analysis, and/or storage for this study take place at nine sites across the United States. Because alcoholism is a complex genetic disorder, the COGA researchers expected that multiple genes would contribute to the risk. In other words, there will be no single “gene for alcoholism” but rather variations in many different genes that together, interacting with the environment, place some people at significantly higher risk for the disease. This genetic and environmental variability (i.e., heterogeneity) makes the task of identifying individual genes difficult. However, the COGA project was designed with these difficulties in mind and incorporated strategies to meet the challenges. This article briefly reviews these strategies and summarizes some of the results already obtained in the ongoing COGA study.

## Study Design

Because of the expected complexity of factors contributing to alcoholism risk, COGA required a large sample size to allow detection of the genetic “signal” through the “noise.” Of particular concern was the likely variability within the sample of both the number and type of genetic and environmental factors contributing to alcoholism risk; therefore, the contribution of any one factor would only account for a small fraction of the variation in risk. The investigators chose a family study design to allow the use of multiple methods of genetic analysis. Systematic recruitment from outpatient and inpatient alcoholism treatment facilities and assessment of families initially was carried out at six sites across the United States, with a seventh site more recently. The study also included a large sample of control families that were randomly selected from the community. For the analyses, the researchers chose a split-sample design—two groups of subjects (i.e., an initial sample and a replication sample) were analyzed independently; this approach allows investigators to examine the reproducibility of the initial study findings.

Because of the complexity of the risk factors for alcoholism and of the disorder itself, the COGA project was designed to gather extensive data from the participants. Although standard diagnostic systems for alcoholism can reliably determine who needs treatment, the diagnostic criteria used in these systems comprise problems in many domains of functioning. This means that two people with the same diagnosis (e.g., alcohol dependence as defined in the *Diagnostic and Statistical Manual of Mental Disorders, Third Edition, Revised* [DSM–III–R] of the American Psychiatric Association [APA] [1987]) may have different sets of symptoms, greatly complicating genetic analyses. Therefore, COGA researchers gathered a detailed psychiatric history of each participant, along with electrophysiological data (electroencephalograms [EEGs] and event-related potentials [ERPs]). These multiple domains of data (described in detail in Begleiter et al. 1995, 1998; Hesselbrock et al. 2001) provide a rich resource for exploring phenotypes related to alcoholism. In addition, they allow analyses under standard diagnostic systems, such as the 4th edition of the DSM (DSM–IV) (APA 1994) and the 10th edition of the *International Classification of Diseases and Related Problems* (ICD–10) of the World Health Organization (WHO) (1992–1994).

The strategies for genetic analyses in the COGA study also had to accommodate the anticipated genetic complexity of alcoholism and the multiple phenotypes that would be collected. Therefore, COGA investigators chose an unbiased survey of the entire genome. For participants from families with three or more alcoholic family members, the investigators conducted genetic analyses using microsatellite markers—DNA regions located across all chromosomes, in which short repeated sequences exist in many variants (i.e., alleles). This process is called genotyping. More than 1.2 million genotypes have been generated on 2,310 people from families of alcoholics and 1,238 people from control families. By monitoring the inheritance patterns of such marker alleles within families with alcoholic members, the investigators could identify chromosomal regions that influence (i.e., show genetic linkage with) certain alcohol-related traits.

The methods used in these genetic analyses and other aspects of the COGA study are described in more detail in the article by Bierut and colleagues, pp. 208–213, in this issue.

## Results of Genetic Analyses

### DNA Regions with Susceptibility Genes

Genetic analyses using the diagnostic criteria for alcohol dependence as the phenotype have revealed regions on several chromosomes that appear to contain genes affecting the risk for alcoholism. The primary analyses were based upon determining the extent of allele sharing among siblings who meet diagnostic criteria for alcoholism. The primary COGA definition of being affected with alcoholism requires a person to meet both DSM–III–R criteria for alcohol dependence and the Feighner criteria (Feighner et al. 1972) for definite alcoholism. If siblings who are alcoholic share more alleles at a marker than would be expected based on chance, this suggests that genes within the chromosomal region containing the marker contribute to the risk of alcoholism.

Analyses of 987 people from 105 families in the initial sample provided evidence that regions on 3 chromosomes contained genes that increase the risk for alcoholism (Reich et al. 1998). The strongest evidence was for regions on chromosomes 1 and 7, with more modest evidence for a region on chromosome 2. The DNA regions identified through these analyses were broad, as is typical for studies of complex genetic diseases, and therefore are likely to contain numerous genes. Much additional work is required to narrow the regions and attempt to determine which specific gene or genes play a role in affecting the risk for alcoholism. Therefore, additional markers within these regions of interest were analyzed in the same people. Subsequent analyses that included the additional markers supported the initial findings (Foroud et al. 2000) but did not narrow the chromosomal regions in which genes influencing alcoholism susceptibility are likely to lie.

The data from the second part of the split sample—the replication sample, which comprised 1,295 people from 157 families—generally supported the initial findings (Foroud et al. 2000). Thus, the replication sample again provided evidence that genes increasing the risk of alcoholism were located in the same regions of chromosomes 1 and 7, albeit with less statistical support. When the initial and replication samples were combined, these chromosomal regions remained the strongest candidates for containing genes influencing the risk of alcoholism. Evidence for the region on chromosome 2 increased with the additional markers in the initial sample, but the replication sample provided no additional evidence for alcoholism susceptibility genes in this chromosomal region. Conversely, the strongest evidence in the replication sample for a region containing genes affecting the risk for alcoholism was on chromosome 3, which had shown no evidence of being linked with alcoholism in the initial sample. Because of the large number of genes that may contribute to the risk for a complex genetic disease such as alcoholism, however, it is not surprising that an independent sample, even one collected by the same group of researchers, might replicate some previously identified genes and also identify some novel alcoholism susceptibility loci.

Foroud and colleagues (2000) also analyzed the combined data set from the initial and replication samples using a more restricted definition of alcoholism as specified in the ICD–10. This restriction greatly reduced the number of sibling pairs in the comparison. The region on chromosome 1 provided the strongest evidence for a susceptibility gene in the combined sample. In addition, this new evaluation detected a region on chromosome 8 that was linked with the risk for alcoholism.

### DNA Regions with Protective Genes

Interestingly, analyses of nonalcoholic sibling pairs in the initial sample produced evidence for a protective region on chromosome 4, in the general vicinity of the alcohol dehydrogenase (*ADH*) genes.^2^ A related analysis, using a technique that treats alcoholism as the extreme of a distribution of an underlying quantitative trait,^3^ showed evidence for linkage to this same region (Williams et al. 1999). This finding suggests that variants of a gene or genes within this region reduced the risk of becoming alcoholic. *ADH* alleles are known to affect the risk for alcoholism; however, the known protective alleles occur at high frequency in Asian populations but are rare in the Caucasian population that makes up most of the COGA sample (Edenberg 2000). Therefore, these analyses may have identified a new protective *ADH* allele or another protective gene located nearby. The number of unaffected sibling pairs genotyped in the replication sample was too small to analyze. Another phenotype that may reflect a protective influence against alcoholism is the maximum number of drinks a person has consumed in a 24-hour period (MAXDRINKS). This phenotype is quantitative and heritable, and a low number of drinks consumed in a 24-hour period may reflect a reduced tolerance for high levels of alcohol. An advantage of a quantitative phenotype is that everyone in a study can contribute to the genetic analysis, not just people who meet diagnostic criteria. Analysis of the MAXDRINK phenotype in both the initial and replication data sets (and in the combined sample) showed the strongest evidence for linkage in the same region of chromosome 4 where the *ADH* genes reside (Saccone et al. 2000). This finding suggests that the gene or genes influencing the MAXDRINKS phenotype may be related to the protective region identified in the unaffected sibling pairs and to protective effects of certain *ADH* alleles (Edenberg 2000).

### DNA Regions Related to Symptoms of Alcoholism

The symptoms used to establish a diagnosis of alcoholism are highly diverse, ranging from more biological symptoms (e.g., tolerance and withdrawal) to more social symptoms (e.g., social and legal problems). Each person diagnosed with alcoholism exhibits a unique mix of those symptoms; therefore, a diagnosis of alcoholism does not reflect a uniform phenotype. This lack of uniformity complicates genetic analyses. Consequently, researchers have constructed other, more defined phenotypes from the data obtained in the COGA interviews. These include an analysis of symptoms related to alcoholism that produced phenotypes which appeared to reflect the severity of alcohol problems. Analysis of these phenotypes provided evidence for a DNA region on chromosome 16 that was associated with an increased risk for more severe alcohol problems (Foroud et al. 1998).

### DNA Regions Associated with Co-Occurring Disorders

Many people in the COGA families of alcoholics also met the DSM–III–R diagnostic criteria for major depressive disorder or depressive syndrome (Nurnberger et al. 2001). Depression alone showed modest evidence of linkage to a region on chromosome 7. The phenotype characterized by co-occurring alcoholism and depression showed evidence of linkage to a region on chromosome 2, primarily in the replication sample. The most interesting finding was for the broad “alcoholism *or* depression” phenotype, with very strong evidence for linkage to the same region of chromosome 1 that was linked to alcoholism alone (Nurnberger et al. 2001). This suggests that a gene or genes within this chromosomal region increase the risk for both alcoholism and depression. (For more information on these analyses, see the article by Nurnberger and colleagues, pp. 233–240, in this issue.)

### DNA Regions Linked with Electrophysiological Measures

The COGA investigators also evaluated electrophysiological variables, such as EEGs and ERPs, from study participants. EEGs measure overall brain activity, whereas ERPs are brain waves elicited in response to specific stimuli (e.g., a light or sound). Analysis of such electrophysiological data may reveal a subset of genes that affect these quantitative, biological phenotypes related to alcoholism (Porjesz et al. 1998, 2002). One component of an ERP is a brain wave called P300, which typically occurs 300 milliseconds after a stimulus. Previous studies had found that a reduced amplitude of the P300 wave is a heritable phenotype that correlates with alcohol dependence and other psychiatric disorders (Porjesz et al. 1998). The genetic analyses of the COGA participants identified four regions, on chromosomes 2, 5, 6, and 13, that appear to contain genes affecting the amplitude of the P300 (Begleiter et al. 1998).

In addition to these findings, recent analyses demonstrate strong evidence for a locus that affects brain wave oscillations as measured by electroencephalography (Porjesz et al. 2002). Thus, a gene or genes that affect brain rhythms lies in a region of chromosome 4 that contains a cluster of genes encoding proteins (i.e., receptors) which interact with the brain chemical gamma-aminobutyric acid (GABA).

### Candidate Genes

COGA researchers have also analyzed candidate genes—genes suspected to play a role in the development of alcoholism based on other studies. Some of these candidate genes encode components of various brain chemical systems that allow communication among nerve cells. Two of these genes are the dopamine D_2_ receptor gene (*DRD_2_*) and a serotonin transporter gene (*HTT)*. However, the analyses found no evidence that *DRD_2_* affected the risk for alcoholism (Edenberg et al. 1998*a*) or that *HTT* was linked to either alcoholism in general or to a more severe form of alcoholism (Edenberg et al. 1998*b*).

## Perspective

Where does the COGA study go from here? The increasing availability of the DNA sequence of the entire human genome and knowledge of variations in that sequence among people are greatly aiding the current phase of the research. Particularly important to the current work is the use of the sequence data to identify which genes are located within the regions that have shown linkage with alcoholism and the other phenotypes examined in the COGA analyses and to identify variations (i.e., polymorphisms) within those genes. Where the available data are incomplete or insufficient, COGA researchers are seeking these polymorphisms themselves. Of particular value are single-nucleotide polymorphisms (SNPs)—sites at which people differ in a single base pair—in or near genes within the regions of interest. COGA investigators are doing additional genotyping of SNPs in and near candidate genes in the regions of linkage for further analysis of linkage and linkage disequilibrium (i.e., the nonrandom association of alleles). This should allow the investigators to greatly narrow the regions and to identify individual genes in which variations affect the risk for alcoholism and the other phenotypes they are studying.

The COGA data set is a rich resource for further research. For example, it has already provided a test of new methods for genetic analysis, as presented at the Genetic Analysis Workshop 11 (Begleiter et al. 1999). In addition, COGA researchers are currently re-interviewing participants as part of a 5-year followup. This strategy will allow the investigators to increase the reliability of the data and to refine the phenotypes, which in turn will enhance the power of the genetic analyses.

Finally, the large number of children and adolescents in the original sample will prove invaluable as these young people pass through the age of greatest risk for developing alcoholism. The value of the COGA data as a national resource for studies of alcoholism should increase with the re-interviews and with the development of new methods for both the determination and analysis of various genotypes. These efforts ultimately are expected to lead to the identification of genes that affect the risk for alcoholism and related phenotypes.

## Footnotes

1 The term “phenotype” refers to any observable characteristic or behavior of an individual.

2 Alcohol dehydrogenase is an enzyme that helps break down alcohol in the body. Several *ADH* genes exist, each of which has several alleles.

3 Quantitative traits are characteristics that are distributed along a continuum across a population, such as height.
